# The mechanism of formation of thin-walled cystic lung cancer

**DOI:** 10.1097/MD.0000000000015031

**Published:** 2019-04-05

**Authors:** Jie Zhang, Hui Deng, Chong Chong Wu, Zhaoyu Wang, Dahai Zhao, Bo Wei, Jing Yuan Zhang, Xinjie Tong, Jie Gao, Lei Pan, Xin Ying Xue

**Affiliations:** a Department of Respiratory and Critical Care Medicine, Beijing Shijitan Hospital, Capital Medical University; b Department of Radiology, General Hospital of PLA, Beijing; c Department of Pathology, Zhoushan Hospital, Zhejiang; d Department of Respiratory and Critical Care Medicine, the Second Hospital of Anhui Medical University, Anhui; e Department of Thoracic Surgery, Beijing Shijitan Hospital, Capital Medical University; f Department of Pathology, General Hospital of PLA, Beijing, China.

**Keywords:** formation mechanism, pathologic characteristics, thin-walled cystic lung cancer

## Abstract

Thin-wall cystic lung cancer is becoming of increasing interest in the study of pulmonary medicine. Consequently, more and more different images and pathologic manifestations have been found. The purpose of this article is to find pathologic characteristics and try to explain the formation mechanism of thin-walled cystic lung cancer.

Sixty-five patients with this special lung cancer were analyzed retrospectively based on the review of medical records, radiologic findings, and pathologic changes.

We found 3 pathologic types: adenocarcinoma, squamous cell carcinoma, and lymphoma. There were 60 cases of adenocarcinoma, 4 cases were squamous cell carcinoma, and only 1 lymphoma. Tumor cells, pulmonary vessels, fibrous tissues, and residual bronchi are the pathologic basis of different image findings.

Thin-walled cystic lung cancers are mostly adenocarcinoma, but other pathologic types can also appear, such as squamous cell carcinoma and lymphoma. We can see that a large amount of fibrous tissues were generated by tumors around the bronchus, resulting in airway stenosis and degeneration. Tumor cells also can invade the bronchial wall and cause structural damage. All these lesions are similar to 1-way valves which can cause gas accumulation in the tumor area and result in thin-walled cystic lung cancer.

## 1 Introduction

Lung cancer is the most common cancer worldwide, with more than 1.8 million new cases in 2012.^[1]^ Lung cancer is the most frequently diagnosed cancer in the United States and the leading cause of cancer death.^[2]^ In the study by Tetsuro Araki et al, pulmonary cysts were seen on computed tomography (CT) in 7.6%.^[3]^ Thin-walled cystic lung cancer was presented as a case report in some literatures.^[4–6]^ It is used to define thick-walled lung adenocarcinoma with a cavity wall thickness of >4 mm, while the thin walled was defined as a space with a wall thickness of 4 mm or less.^[7]^ We try to explain the formation mechanism of thin-walled cystic lung cancer by analyzing imaging and pathologic characteristics.

## 2 Materials and methods

We collected information on 65 cases of thin-wall cystic lung cancer treated in 5 hospitals in Beijing from 2015 to 2018. The diagnosis of this disease was based on radiologic findings and biopsy after surgical resection or bronchoscopic biopsy. All patients underwent chest CT, 54 patients underwent pulmonary lobectomy or wedge excision and 11 patients underwent chemotherapy because of metastasis. We retrospectively analyzed these cases after reviewing their radiologic findings, pathologic types, and medical records. Sixty-four multidetector CT with volumetric acquisition with slice thickness of 1.25 or 1.5 mm for all patients. This study was conducted in compliance with the institutional policy regarding the protection of patients’ confidential information and was approved by the Research Ethics Committee of Beijing Shijitan Hospital affiliated to Capital Medical University. All procedures were carried out in accordance with the approved guidelines of Beijing Shijitan Hospital affiliated with Capital Medical University.

## 3 Results

There were 44 male (67.7%) and 21 female (32.3%) patients in our study (Table 1). Their ages ranged from 33 to 78 years, and 56.9% of them were below 60 years old. Moreover, 61.5% of them said they had no history of smoking.

**Table 1:**
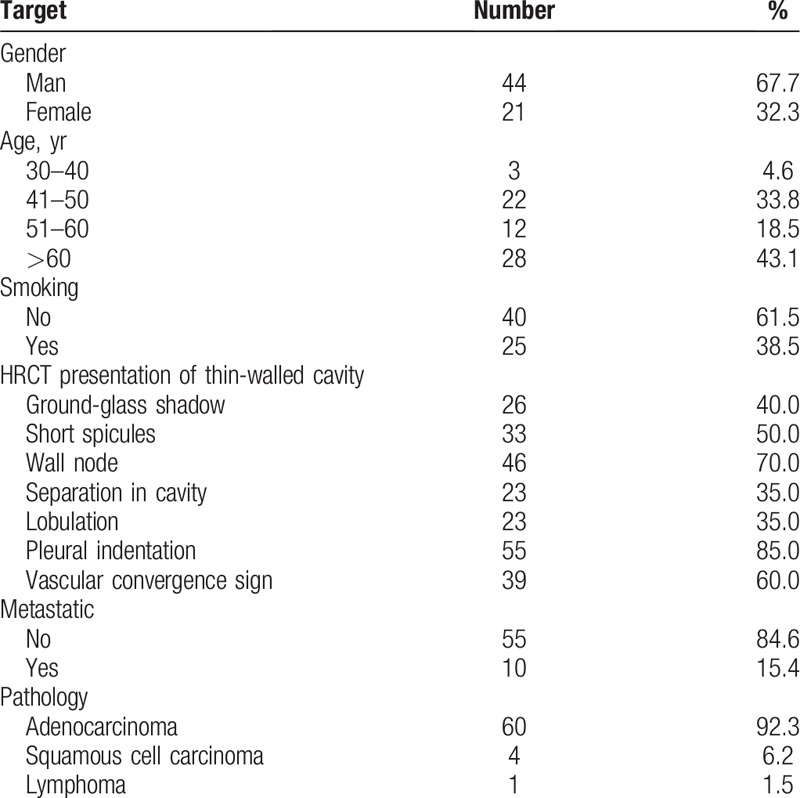
Clinical characteristics of 65 patients with thin-walled cystic lung cancer.

**Figure 1:**
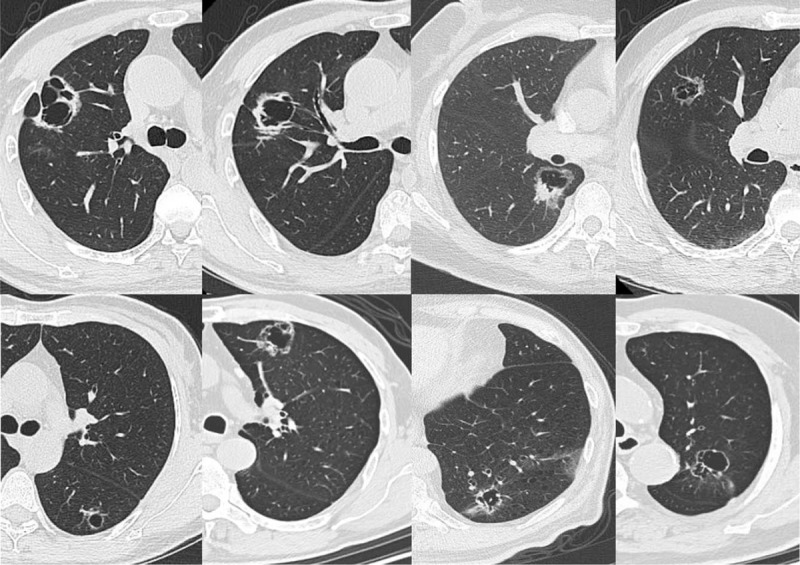
Chest computed tomography presentations of solitary thin-wall cystic lung cancer.

The CT manifestations of thin-wall cystic lung cancer are shown in Figure 1. The wall thickness of cysts ranges from about 1 to 4 mm. Each of them displayed one or more suspected malignant signs of lung cancer, including asymmetric thickening of the wall (100%), separation in cysts (86.3%), wall-node (70.0%), irregular margin (65.6%), and ground-glass opacity around cystic lung cancer (40.0%).

A 48-year-old female patient presented at our outpatient department with a cough and sputum due to a cold. A 5.7 × 4.8 cm cystic lesion was visible in the right lower lobe on CT. There was no microbiologic or histopathologic evidence of TB. This patient was empirically treated with ATT for X-ray and CT finding suggestive of TB. Isoniazid, Rifampicin, and Pyrazinamide combination therapy was administered to treat mycobacterium tuberculosis for 3 months, but there were no obvious changes in the lesion. The outer wall, lobule, and inner wall are not smooth on CT. Due to these signs, the possibility of malignant lesions was not ruled out. Therefore, CT-guided percutaneous biopsy (CT-PB) was used to confirm the diagnosis. Pathologic findings were in favor of marginal zone lymphoma.

**Figure 2:**
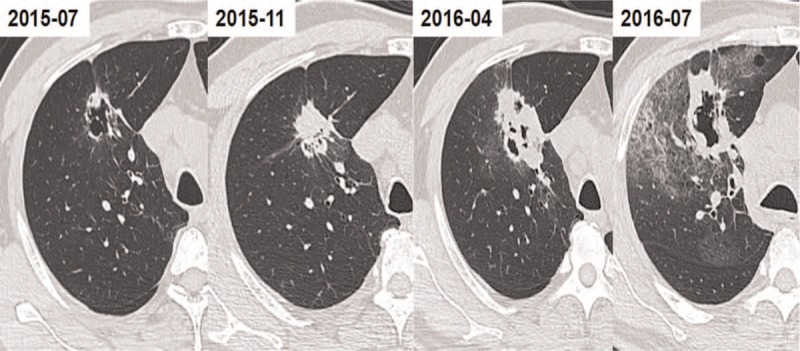
The possible evolution of the cavity.

**Figure 3:**
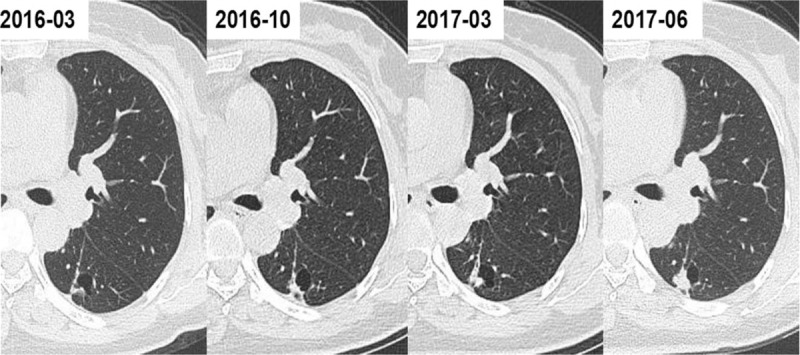
The gradually thickened cavity wall.

A 52-year-old patient who had occasional bloody sputum and a history of smoking also got admitted in our hospital. CT showed a cystic lesion in the upper right lung, which could be possible lung cancer. PET/CT showed high metabolic lesion with multiple highly metabolic lymph nodes in the neck and right mediastinum. The pathologic result of lymph node biopsy is low differentiated adenocarcinoma. He was treated with Pemetrexed (0.9 g QD). Imaging examinations showed an increasingly smaller cyst and increasing parenchyma for <3 months. The last imaging analysis showed formation of a new cavity with a wall thickness of >4 mm (Fig. 2). On the contrary, in another patient, the growth rate of tumor cells was slower, and the cavity still existed for more than a year (Fig. 3).

**Figure 4:**
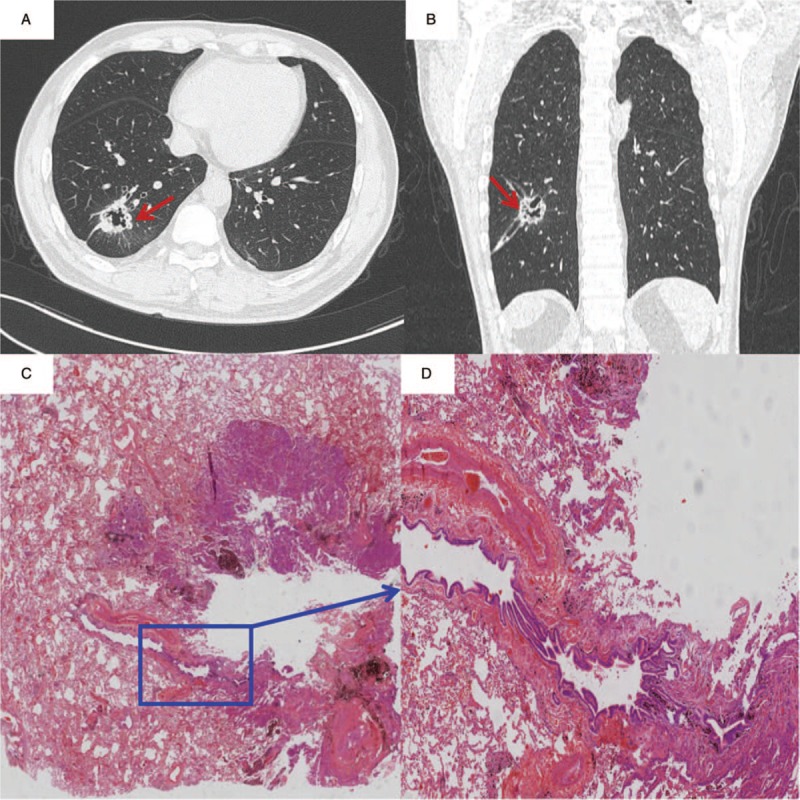
On the computed tomography film of a 46-year-old male patient, we can see tracheal and vascular structure on the wall of the cavity (A and B). On the pathologic scanning, we can clearly see the boundary of the tumor (C) and see that the bronchus is connected to the cavity (D).

A 64-year-old male was admitted to the hospital with chest tightness, cough, and expectoration. Pulmonary CT showed a cyst in his left upper lobe. The cystic wall was uneven and lobulated. Vascular bundle sign and segmental bronchi pass through it. Finally, the left lung lobectomy was performed. The pathologic result was moderately differentiated adenocarcinoma. By scanning the pathologic slices, we can clearly see the boundary of the tumor, the boundary was irregular, and we could see that the damaged bronchus leads to the cavity. The bronchus leads to the cystic cavity, which causes the cavity to become larger, and gradually form thin-walled cystic lung cancer. This should illustrate the 1-way valve mechanism (Fig. 4).

**Figure 5:**
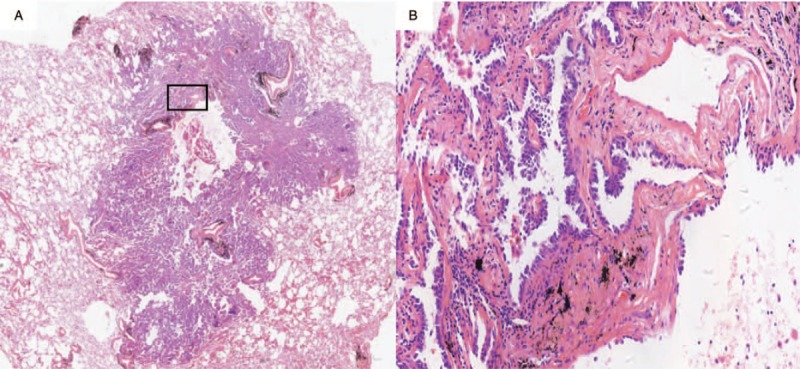
The wall of the tumor cells mainly contains some blood vessels and hyperplastic fibrous tissue.

Pathologic scan of thin-walled cavity adenocarcinoma showed uneven wall thickness and clear demarcation with normal tissue. We can see tumor cells with large number of proliferating fibrous tissues and blood vessels of nutritional tumors (Fig. 5). These fibrous tissues can lead to the destruction of normal lung structure and abnormal traction of lung tissue.

## 4 Discussion

Cavities are frequently seen manifestations in a wide variety of pathologic processes involving the lung. Cavities were used to identify as clinical subentity in squamous cell lung cancer.^[8]^ Recently, cases of thin-walled cystic lung cancer have also been increasingly reported; however, the number of cases is small and the pathologic results were incomplete. This study mainly analyzes the imaging findings and the possible disease mechanism by analyzing pathologic results. Thin-wall cystic lung cancer is most common in middle and high differentiated adenocarcinomas. Qi and Zhang^[9]^ also reported 16 cases of adenocarcinoma. Xue et al^[10]^ reported thin-wall cystic lesions detected in 15 patients with moderately or well-differentiated adenocarcinoma. In our study, the presenting pathologies were not limited to adenocarcinoma, it can be squamous cell carcinoma or lymphoma. Maybe other types can be found in later work. These suggest that the formation of thin-wall cystic lung cancer may not be related to pathologic type. The mechanism of the formation of thin-wall cavity is not clear at present. Through studying pathology, we speculate that the possible mechanism may be explained as follows.

A large amount of fibrous tissue generated by tumor around the bronchus, resulting in airway stenosis and degeneration. All these lesions are similar to 1-way valves, which can cause gas accumulation in the tumor area and result in thin-walled cystic lung cancer. We think that when tumor cells grow slowly, thin-walled cavities are easy to form. However, faster growth speed will lead to necrosis and form thick-walled cavity. A check-valve mechanism is widely accepted.^[11–13]^ The check valve is difficult to observe in pathologic sections, but we successfully found the pathologic evidence of bronchial opening in the cysts.

From pathologic understanding of the characteristics of the disease, we can accurately judge if the tumor is benign or malignant when we see similar images. As sample size was small, no statistical analysis was done. The next step is to prepare and improve the follow-up of patients, and further discussion about metastasis, prognosis, and pathologic types.

## 5 Conclusion

In this study, the pathology results are used to explain the possible mechanism of the formation of thin-walled cavity. The authors think that the basic formation of the 1-way valve may be associated with growth rate of the tumor, but the pathologic type may not be directly associated with the 1-way valve. We also introduced the pathologic basis of imaging findings in detail, which can help us better understand this disease.

## Author contributions

**Conceptualization:** Bo Wei, Xin Ying Xue.

**Data curation:** Hui Deng, Chong Chong Wu, Dahai Zhao, Jing Yuan Zhang, Jie Gao, Lei Pan.

**Formal analysis:** Hui Deng.

**Funding acquisition:** Xin Ying Xue.

**Investigation:** Dahai Zhao.

**Project administration:** Bo Wei.

**Resources:** Chong Chong Wu, Zhaoyu Wang, Jing Yuan Zhang, Jie Gao, Lei Pan.

**Software:** Xinjie Tong.

**Supervision:** Chong Chong Wu, Zhaoyu Wang, Lei Pan, Xin Ying Xue.

**Validation:** Xinjie Tong, Xin Ying Xue.

**Writing – Original Draft:** Jie Zhang, Hui Deng.

**Writing – Review & Editing:** Hui Deng.
